# Where the College of Nursing Stands

**Published:** 1918-10-05

**Authors:** Gertrude Cowlin

**Affiliations:** Organising Secretary, the College of Nursing, Limited by Guarantee.


### THE HIGHER EDUCATION OF NURSES.*

### Where the College of Nursing Stands.

By GERTRUDE COWLIN, Organising Secretary, the College of Nursing, Limited by Guarantee.

In spite of the work of the already organised nurses' societies which have worked on behalf of nurses for so many years, the nursing profession upon the outbreak of war was unable officially to define a trained nurse, and there was no comprehensive register of trained nurses or of training schools. Had such an organisation as the College of Nursing existed before 1914, it would have been possible by referring to the Register to have placed all the *bona-fide* trained nurses in the posts of authority due only to the trained woman, and much mismanagement and friction would have been avoided. In his capacity of chairman of the British Red Cross Society, Sir Arthur Stanley saw how badly we needed some central authority such as other professions have, an authority composed of nurses and managed by nurses; and since it should of necessity be also an educational headquarters, Sir Arthur, anxious to know what measure of support he would secure from those responsible for the education of nurses, promptly wrote to the training-schools and appealed to the leading matrons in London and the provinces. His appeal met with so much encouragement that he decided to launch his project at the earliest possible date as a piece of National Service, and so the College was established. The matrons who decided to give Sir Arthur Stanley their support were those of whom many had already belonged to some of the existing organised nurse societies; but, feeling that a new age demanded new methods, readily undertook to support Sir Arthur's scheme, and decided that one of the primary objects of the College should be to obtain State registration for nurses, and a Bill was accordingly drafted. If we can support our claim for State registration by producing a voluntary Register of Trained Nurses, unanimous in their wish for legal status, our chances of obtaining it are immeasurably greater than if we went to Parliament without it; so this was promptly done, and in two years we have compiled a register of 10,000 nurses, which we hope to present to Parliament at an early date.

### Reasons for Joining the College.

Here I might give you a few of my own reasons for joining the College of Nursing :—

1. It stands for State registration, and in the Bill promoted by the College it is guaranteed that two-thirds of the General Council controlling this registration shall be elected by the members—i.e. the nurses on the Register, and that of this number five-sixths must themselves be nurses. Please note this proportion, two-thirds, so that no measure will be allowed in which the voice of the nurse has not predominated.

2. It stands for even more than State registration—for the higher education of nurses and improved conditions of nursing throughout the country. This has been realised already, and conditions in certain hospitals changed in order to bring them up to the standards laid down by the College.

3. It is a central organisation to form a medium whereby nurses will at last be able to organise their own affairs, so that we may move forward as a self-governing whole.

4. It proposes to regulate standards of training by (*a*) a three years' general training; (*b*) one-portal examination; (*c*) a definite curriculum in a recognised training-school.

5. It stands to carry on the highest traditions and ideals of nursing which have been handed down to us, and which no conditions which have formerly prevailed have been able to eradicate.

### The Training and Equipment of the Nurse.

How is the College going to help to equip the nurse for increasing responsibilities? Some nurses receive a training which equips them for all we desire of them, but there are other nurses who have never had a chance of anything but the most meagre experience, and who have been victims of a most haphazard training. How can we alter this situation? The College of Nursing has for its object the introduction into all training-schools of a uniform curriculum, so that all probationers, in whatever institution, will be studying the same subjects at the same stages of their training. In the case of training-schools which are unable to give experience in any one branch of nursing, and which may be uncontrollable for many reasons—as, for instance, the particular needs of any one locality—the College will encourage a scheme of affiliation or reciprocity with other institutions.

Some institutions, small or poor hospitals supported by voluntary contributions, would find it a heavy tax on their funds to provide instructors. Such cases could be met by the College having a staff of trained teachers, who could visit institutions periodically and give a course of instruction in the subject or subjects required.

On the other hand, it will always be desirable for institutions to have resident instructors, and what the College has to do now is to help to qualify nurses for this particular branch of work. Studentships have been offered by the College of Nursing, tenable at King's College, for women, and on October 1 three successful nurse candidates go into residence for a year's course in the teaching of Nursing Methods.

### Opportunities for Graduate Nurses.

The programme of the College also is to include the establishment of scholarships for graduate nurses wishing to specialise after training in any branch of public service—i.e. travelling scholarships, that the methods of other countries may be observed; courses in post-graduate study, that nurses may be kept up to date in the latest methods; courses in administration; courses in the teaching of nursing methods. It hopes also to be able to make grants for equipment or educational advantages to smaller training-schools; offers of free legal advice to its members; and to give assistance to its members who may be financially embarrassed through sickness.

The establishment of branches or Local Centres throughout England has met with so much encouragement that there are already now twelve flourishing Centres doing excellent work. The object of the Centre is to consolidate the members of the College and to give each member an opportunity to contribute towards the organisation of her profession. You will understand how important it is for the headquarters to keep in touch with the branches in order to ensure the mutual help and support necessary for uniform development. A Standing Committee therefore has been formed, upon which the local representative of each branch serves, and which is dealing entirely with the difficulties and problems touching the Local Centres. The progress of the various Centres is reported by the representatives at this committee, and if there is any particular subject any branch wishes to have discussed it is included in the agenda of the meeting.

* Address at a meeting held at Addenbrooke's Hospital, Cambridge, on September 11.

